# Generation of Diversity in *Streptococcus mutans* Genes Demonstrated by MLST

**DOI:** 10.1371/journal.pone.0009073

**Published:** 2010-02-05

**Authors:** Thuy Do, Steven C. Gilbert, Douglas Clark, Farida Ali, Clarissa C. Fatturi Parolo, Marisa Maltz, Roy R. Russell, Peter Holbrook, William G. Wade, David Beighton

**Affiliations:** 1 Infection Research Group, Dental Institute, King's College London, London, United Kingdom; 2 Faculty of Dentistry, Department of Social and Preventive Dentistry, Federal University of Rio Grande do Sul, Porto Alegre, Brazil; 3 School of Dental Sciences, Newcastle University, Newcastle upon Tyne, United Kingdom; 4 Faculty of Odontology, University of Iceland, Reykjavik, Iceland; Duke University Medical Center, United States of America

## Abstract

*Streptococcus mutans*, consisting of serotypes c, e, f and k, is an oral aciduric organism associated with the initiation and progression of dental caries. A total of 135 independent *Streptococcus mutans* strains from caries-free and caries-active subjects isolated from various geographical locations were examined in two versions of an MLST scheme consisting of either 6 housekeeping genes [*accC* (acetyl-CoA carboxylase biotin carboxylase subunit), *gki* (glucokinase), *lepA* (GTP-binding protein), *recP* (transketolase), *sodA* (superoxide dismutase), and *tyrS* (tyrosyl-tRNA synthetase)] or the housekeeping genes supplemented with 2 extracellular putative virulence genes [*gtfB* (glucosyltransferase B) and *spaP* (surface protein antigen I/II)] to increase sequence type diversity. The number of alleles found varied between 20 (*lepA*) and 37 (*spaP*). Overall, 121 sequence types (STs) were defined using the housekeeping genes alone and 122 with all genes. However π, nucleotide diversity per site, was low for all loci being in the range 0.019–0.007. The virulence genes exhibited the greatest nucleotide diversity and the recombination/mutation ratio was 0.67 [95% confidence interval 0.3–1.15] compared to 8.3 [95% confidence interval 5.0–14.5] for the 6 concatenated housekeeping genes alone. The ML trees generated for individual MLST loci were significantly incongruent and not significantly different from random trees. Analysis using ClonalFrame indicated that the majority of isolates were singletons and no evidence for a clonal structure or evidence to support serotype c strains as the ancestral *S. mutans* strain was apparent. There was also no evidence of a geographical distribution of individual isolates or that particular isolate clusters were associated with caries. The overall low sequence diversity suggests that *S. mutans* is a newly emerged species which has not accumulated large numbers of mutations but those that have occurred have been shuffled as a consequence of intra-species recombination generating genotypes which can be readily distinguished by sequence analysis.

## Introduction

*Streptococcus mutans* is the major species of mutans streptococci, isolated from the human oral cavity [1], [2], being acquired before the emergence of the primary dentition [3] and which proliferates in an oral environment conducive to the initiation and progression of carious lesions [4]. *S. mutans* may be identified using simple biochemical tests [5] or with PCR-based methods in which the presence of *S. mutans*-specific genes is determined [6]–[8]. Isolates of *S. mutans* can be assigned to one of four serotypes (c, e, f and k) based on their reactions with serotype-specific antisera [9]–[11] or by the detection of serotype-specific genes coding for glycosyltransferases [12], [13].

A range of methods have been applied to *S. mutans* typing, one of the earliest of which was based on susceptibility to bacteriocins [14], [15] but was found to lack reproducibility and was not readily transferred between laboratories. This approach was superseded by restriction fragment length polymorphism (RFLP) schemes based on the comparison of whole genomic DNA [16]. However these patterns, while highly discriminatory, were difficult to analyse due to the high number of DNA fragments. These patterns were simplified by the application of ribotyping [17] and by the use of pulsed-field gel electrophoresis with rare-cutting endonucleases to fragment the genomic DNA [18]. PCR-based methods such as arbitrary primed PCR [AP-PCR] [19]–[22] provided adequate discrimination but was subject to the inter-laboratory reproducibility difficulties typical of PCR-based typing schemes. The primary conclusion of the various studies using these schemes was that *S. mutans* was a diverse species to the extent that independent subjects rarely shared the same genotypes. However, *S. mutans* genotypes were passed from mothers, or prime carers, to their offspring, although other genotypes of exogenous origin would also be present. Although the different approaches used to date have yielded interesting data, a comprehensive survey of the population biology of this species requires a portable, reproducible scheme.

Multilocus sequence typing (MLST) was developed and first described for *Neisseria meningitidis* [23] and then has been successfully applied to many pathogens such as *Streptococcus pneumoniae* [24], *Streptococcus pyogenes* [25], and *Campylobacter jejuni* [26]. More recently this approach has been applied to commensal bacteria including *Lactobacillus plantarum* [27], *Lactobacillus casei* [28], *Streptococcus salivarius* [29] and *Streptococcus oralis* [30]. The major advantage of this technique over others is that the allelic profiles of the isolates can easily be compared among different laboratories via the Internet [31]. MLST schemes are usually based on partial sequences of house keeping genes which enable the data to be used for phylogenetic analysis since the house keeping genes will not be subject to immune selection [32], [33] which might direct and accelerate genetic change such that phylogenetic relationships are distorted. However, in order to increase the discriminatory ability of MLST schemes, putative virulence determinants have been successfully included [34]. An MLST scheme developed for *S. mutans*, incorporating only house-keeping genes, and applied to a collection of strains isolated in Japan and Finland suggested that a strain possessing the serotype c antigen was the ancestral strain for all *S. mutans* [35]. Here, using a different set of loci, we confirm the diversity of independent *S. mutans* isolates but provide evidence that a *S. mutans* strain possessing the serotype c antigen is not the ancestral strain of all *S. mutans* serotypes (c, e, f, and k). We also demonstrated that two putative virulence determinants, glucosyltransferase B [*gtfB*] and surface protein antigen I/II [*spaP*], were the most diverse of the loci investigated here and that, unlike the house-keeping genes the recombination/mutation ratios were <1 indicating that mutation and not recombination was the driver of diversity in these genes.

## Materials and Methods

### Bacterial Strains

A total of 135 *S. mutans* isolates, including strain UA159 whose genome has been sequenced [36] were included in this study. The strains were isolated from individual subjects in different countries including Japan, Brazil, South Africa, USA, UK, Turkey, New Guinea, Iceland and China (Table S1). The isolates were cultured anaerobically on Columbia agar (Oxoid, UK) supplemented with 5 % (v/v) horse blood and stored at -80°C in glycerol broth. The identity of each isolate was confirmed by partial 16S rDNA gene sequence analysis. The isolates were serotyped, except UA159, using PCR-based methods [11], [12].

### PCR Amplification and Gene Sequencing

Isolates were grown anaerobically for 24 h on Columbia agar and colonies were removed using sterile loops, suspended in 50 µl of sterile deionized water, vortexed and heated at 100°C for 10 min (Microtherm microtube incubator; Camlab, UK) with shaking at 50 rpm to disrupt the cells. The samples were centrifuged at 13,000 rpm for 10 sec and the supernatants containing extracted bacterial DNA were used directly as templates in PCRs.

Partial gene sequences for six housekeeping genes [*accC* (acetyl-CoA carboxylase biotin carboxylase subunit), *gki* (glucokinase), *lepA* (GTP-binding protein), *recP* (transketolase), *sodA* (superoxide dismutase), and *tyrS* (tyrosyl-tRNA synthetase)] and two extracellular putative virulence-associated genes [*gtfB* (glucosyltransferase B) and *spaP* (surface protein antigen I/II)] were obtained. These house keeping genes were selected on the basis that only a single copy of each was present in the genome and that they were evenly spaced around the completed genome of *S. mutans* strain UA159 [36]. The primer sequences used for the initial amplification and the primers, forward and reverse, used for amplicon sequencing are shown in Table 1.

**Table 1:** Primers sequences used to amplify and sequence each locus.

Locus	Gene product	PCR primer sequences5′→3′		Sequencing primer sequences5′→3′
*accC*	acetyl-CoA carboxylase- biotin carbolylase subunit	*accC-F* *accC-R*	TGTGCGTATCATTC GCATCACCCATTTTACCACG	TTGTGACTGTTGCAGT GCAATGAACAATCACG
*gki*	glucokinase	*gki-F* *gki-R*	GTCCTCTATCAAGCATCG TATCACCTGCCTGTGCAGCA	GTCCTCTATCAAGCATCG ACTGCTCACATCATCACC
*lepA*	GTP-binding protein	*lepA-F* *lepA-R*	CTCTATTATTGCCC TACATCACCCGTTG	ATGGGAAGTCAACGCT GACCATACCATCCAT
*recP*	transketolase	*recP-F* *recP-R*	AAGTCTAAGTCGGGTC TGACCAAGCATCGTAAGCAG	CTGTTGGTTTGGCTCA TGACCAAGCATCGTAAGCAG
*sodA*	superoxide dismutase	*sodA-F* *sodA-R*	GGCTATTCTTTTACCAG GGCATAAAGACGAGCAACAG	GCTTATGACGCACTTGAACC GGCATAAAGACGAGCAACAG
*tyrS*	tyrosyl-tRNA synthetase	*tyrS-F* *tyrS-R*	CCAACAGCAGATAG CCACGACGAATAAC	GAGGCATTTACAATTGGC AGCCCCGTTTTGAAGA
*gtfB*	glucosyltransferase	*gtfB-F* *gtfB-R*	GGACAAGAAAGTGCGT AGTTTGATTGGTCGGG	CTGCTGTGATGACTT TACCAACTTTCGGCTGTC
*spaP*	surface protein antigen	*spaP-F* *spaP-R*	AGTGCGAGTAAGGAAG TTCACCGCTGCCAAAT	TGCTAAGTCTGCTGGTGTCA GCT(CT)GATAGTCTGCTTCG

The initial PCRs for all genes were performed with the following conditions: 5 min denaturation at 96°C, followed by 30 cycles of 30 sec at 95°C, 30 sec at 49°C and 90 sec at 72°C. After the last cycle, the samples were incubated at 72°C for 5 min and held at 4°C. The 25 µl-reaction mixture consisted of 22.5 µl of 1.1x ReddyMix PCR master mix (1.5 mM MgCl_2_) (ABgene, UK), 0.5 µl of each primer (10 µM initial concentration) and 1.5 µl of the DNA template. The PCR amplicons were cleaned and purified using microClean (Microzone Ltd, UK). Both strands of the resulting amplicons were sequenced in reactions containing 2 µl of PCR product, 0.5 µl of BigDye v3.0 (Applied Biosystems, UK), 1.75 µl of 5x solution buffer (Applied Biosystems, UK), 1.75 µl of sterile UHQ water and 4 µl of primer (3 pmol). The cycling protocol and cleaning of sequencing reaction products were as described in the manufacturer's protocols and sequencing was performed using an ABI 3730xl DNA Analyzer (Applied Biosystems, UK).

### Data Analysis

The consensus sequence for each gene fragment was determined by alignment of the forward and reverse sequences using BioEdit version 7.0.5.3 (http://www.mbio.ncsu.edu/BioEdit/bioedit.html). The coding sequences used for the housekeeping gene were read in-frame. Allele sequences that differed from each other by one or more polymorphisms were assigned a unique allele number in the order of discovery. Each unique allelic profile, as defined by the allele numbers of the 8 loci (*accC*, *gki*, *gtfB*, *lepA*, *recP*, *sodA*, *tyrS* and *spaP*) was assigned a sequence type (ST) number according to the previously described methodology [23].

The G+C content of each locus, the number of polymorphic sites, average number of synonymous and non-synonymous sites, average nonsynonymous/synonymous rate ratio (*dN*/*dS*), Tajima's D, the nucleotide diversity per site (π) and the average number of nucleotide differences per site (θ) were calculated using DnaSP version 4.0 [37].

Evidence for recombination between STs of each allele was investigated using a number of different approaches. Split decomposition trees were constructed with 1,000 bootstrap replicates based on parsimony splits as implemented in SplitsTree 4.0 [38]. The resulting trees, for individual loci and for concatenated sequences of all 8 loci, were analysed using the pairwise homophasy index (PHI) test [39] to identify alleles with significant evidence of recombination. Evidence was also obtained by analyzing all STs with the algorithms implemented in the RDP program [40]. We assumed that evidence for recombination would be accepted if significant (p<0.001) evidence for the same recombination event was demonstrated with at least 2 tests implemented in the RDP program. Evidence for recombination was also sought by the construction of Maximum-likelihood (ML) phylogenetic trees using PAUP version 4 beta 10 [41]. ML trees for each of the 8 genes were computed and compared using the Shimodaira-Hasegawa (S-H) test to identify significant differences between the tree topologies (differences in log likelihood, Δ-ln *L*). In a clonal population each phylogenetic tree should be congruent and there should not be significant differences in likelihood [42], [43]. To assess the extent of congruence among the ML trees, randomisation tests were performed [44], in which the Δ–ln *L* values for each of the 8 genes were compared to the equivalent values computed for 200 random trees created from each gene. We also assessed each locus for recombination breakpoints using the genetic algorithm detection (GARD) method [45]. The alleles were also tested for positive and negative selection of individual codons using the single likelihood ancestor counting (SLAC) method [46] with both the General Reversible (REV) and HKY85 models of nucleotide substitution.

The degree of clonality was estimated by calculating the index of association (I_A_) between all STs as implemented in START2 (http://pubmlst.org/software/analysis/start2/) [47]. Potential clonal complexes were tentatively identified using the concatenated sequences of only the six housekeeping genes by constructing Neighbour-Joining tree [48] based on the distance and calculating bootstrap values [49] to determine the reliability of branch points and clusters identified in the consensus NJ tree. We next used eBURST (http://eburst.mlst.net/default.asp) as implemented in START2 [47] to identify potential clonal complexes and founders. ClonalFrame [50] was also used to investigate the population structure of *S. mutans*. ClonalFrame is a method for using multilocus sequence data to infer the clonal relationships of bacteria and assumes that recombination events were introduced at a constant rate of substitution to a contiguous region of sequence. The model is reported to have advantages over other methods, including bootstrapping and eBURST, for subdividing recombinogenic bacteria [51]. ClonalFrame was also used to calculate the recombination/mutation (r/m) ratios of the concatenated housekeeping genes and of the concatenated sequences of *gtfB* and *spaP* as it was expected that differences might occur between the intracellular housekeeping genes and the intracellular putative virulence determinants.

## Results

### General Characterization

A total of 122 STs were identified amongst the 135 *S. mutans* isolates when all loci, including the potential virulence determinants, were used to assign STs to individual strains. (Table S1). Assigning isolates to STs on the basis of only the six house keeping genes reduced the number of identified STs to 121. The general characteristics of each locus are shown in Table 2. The G+C mol% content varied between 34.0% for the partial *gtfB* sequence to 43.1% for the *gki* sequence. The virulence genes *gtf*B and *spa*A had 10.37 and 7.99% of all sites polymorphic and these were greater than the level of polymorphisms observed in the housekeeping genes, except for *recP* (8.02%). The number of alleles and polymorphic sites identified per locus was overall low which was reflected in the low θ and π values calculated for each gene. The dN/dS values were all <1 indicating that all loci were subject to stabilizing selection. However, in both putative virulence genes and two of the housekeeping genes the number of non-synonymous changes was greater than the number of synonymous changes.

**Table 2:** Characteristics of loci of the 135 independent *S. mutans* strains.

Locus	Fragment size (bp)	No of alleles	H [±SE]	G+C mol	No. polymorphic sites (%)	Synonymous changes	Nonsynonymous changes	*dN/dS*	Tajima's D test	θ	π
*spaP*	513	37	0.865±0.02	0.396	41 (7.99)	18	21	0.130	−2.172*	0.015	0.004
*gtfB*	453	34	0.883±0.02	0.340	47 (10.37)	12	32	0.495	−1.965*	0.019	0.007
*accC*	462	23	0.540±0.05	0.404	21 (4.55)	11	9	0.176	−2.149*	0.008	0.002
*gki*	426	31	0.860±0.02	0.431	26 (6.10)	12	12	0.196	−1.570	0.012	0.005
*lepA*	441	20	0.689±0.03	0.417	15 (3.40)	7	6	0.068	−1.363	0.007	0.003
*recP*	474	37	0.905±0.02	0.405	38 (8.02)	18	22	0.306	−1.729	0.015	0.007
*sodA*	492	21	0.880±0.02	0.413	21 (4.26)	10	12	0.127	−1.067	0.008	0.005
*tyrS*	513	32	0.881±0.02	0.385	23 (4.48)	15	8	0.041	0.082	0.008	0.008

π, nucleotide diversity per site and θ, average number of nucleotide differences per site and dN/dS  =  Mean non-synonymous substitutions per non-synonymous site (dN)/Mean synonymous substitutions per synonymous site (dS).

*P-value for Tajima's D test <0.05.

### Evidence for Recombination

**Figure 1:**
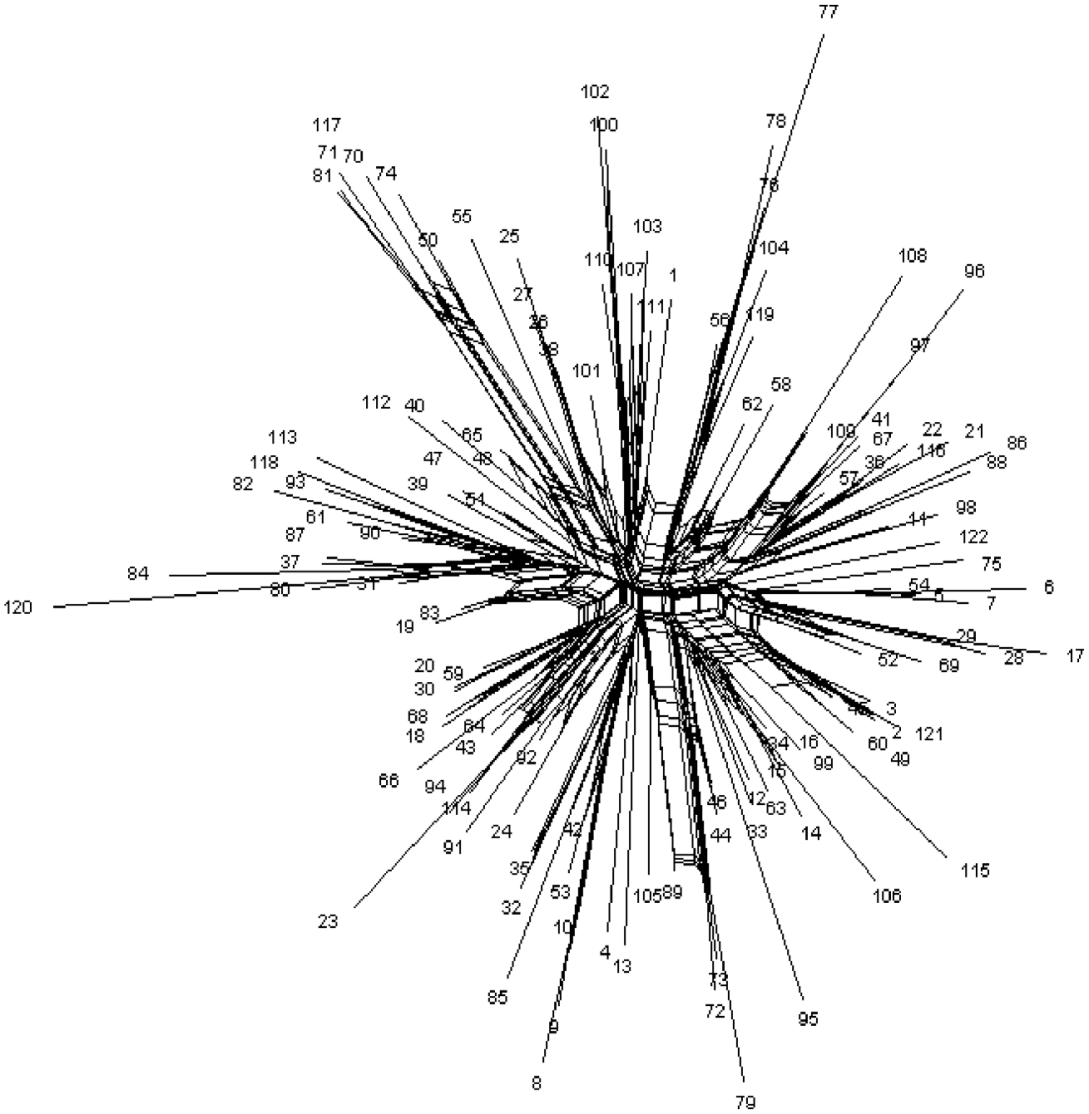
Split decomposition analysis showed a bushy network. NeighbourNet graph of the concatenated sequences of all STs based on the 8 loci with significant (p = 2.50×10^−4^) evidence of recombination with PHI test [39] constructed in SplitsTree v4.0.

Evidence for recombination in the loci was investigated using a number of different approaches. The I_A_, based on a single representative of each sequence type, was 0.4379 (p<0.001) suggesting an underlying clonal population structure. To test further for evidence of recombination, we used the programme SplitsTrees on each locus separately and on the concatenated sequences of all STs. Only *tyr*S exhibited significant evidence of recombination (p = 0.021), in all cases parallelogram formation, indicative of some recombination, was evident (Figure S1). However, when the concatenated sequences of all STs were investigated**,** evidence of significant recombination (p = 2.50×10^−4^) was found and even with the *tyrS* sequence data removed significant evidence for recombination was found (p = 0.016). The split decomposition analysis showed a bushy network structure with extensive parallelogram formation indicative of pervasive homologous recombination (Figure 1).

The sites of recombination could not be identified reliably using the RDP suite with the above criteria. The same result was obtained with the GARD analysis; there was no evidence of recombination in any of the 8 genes. Further the SLAC analysis, with either REV or HKY85 models of nucleotide substitution, failed to identify any positively selected sites in any of the genes, including the two putative virulence genes *gtfB* and *spaP* although 2 sites in *gtfB* and 1 in *spaP* were identified as being negatively selected.

The S-H test for congruence demonstrated that all ML trees generated for individual MLST loci were significantly incongruent (Table 3) and comparison of the Δ*-lnL* values for individual ML trees indicated that these were not significantly different from randomly generated trees suggesting a non-clonal population structure. However, it may be that the overall lack of variation in the sequence data, indicated by the low θ and π values, resulted in apparent incongruence when previous results were suggestive of a clonal structure.

**Table 3:** Tests for congruence among *S. mutans* ML gene trees and random trees (P<0.05 for all *–lnL* values).

Locus	*- lnL*	Δ*-lnL*	Δ*-lnL* of random trees
*accC*	810.531	104.608–113.170	104.235–114.36
*gki*	839.729	280.493–313.321	288.165–331.800
*gdh*	999.510	491.605–565.530	488.512–557.072
*lepa*	745.046	121.764–153.250	115.793–157.814
*recP*	1043.187	419.041–467.968	386.952–466.022
*sodA*	863.037	283.370–436.696	384.652–437.677
*spaP*	1034.739	255.437–327.771	289.294–326.024
*tyrS*	951.961	603.343–708.729	592.097–684.276

To determine whether recombination or mutation was the main driver of incongruence in these trees the r/m ratio was calculated and found to be 8.3 [95% confidence interval 5.0–14.5] for the 6 concatenated housekeeping genes alone. This indicates that recombination and not mutation is the major cause of incongruence between the trees of the housekeeping genes: recombination events amplify and shuffle the diversity arising from mutational events. However, the r/m value for the two virulence genes concatenated was 0.67 [95% confidence interval 0.3–1.15] indicating that mutation was more responsible than recombination for the diversity observed in *gtfB* and *spaP*.

### Inter-Relatedness of Isolates

The NJ tree constructed using the concatenated sequences of the six housekeeping loci did not identify any major branches with acceptable (≥75%) bootstrap values (Figure S2); the majority of bootstrap values were <25% indicating that the topology of the consensus tree was inherently unreliable. Analysis with eBURST identified a limited number of simple complexes with the following STs linked: 65 and 48, 2 and 121, 108 and 109, 22 and 36, 15 and 16 and 26, 27 and 38 (Figure S3). No founders were identified.

**Figure 2:**
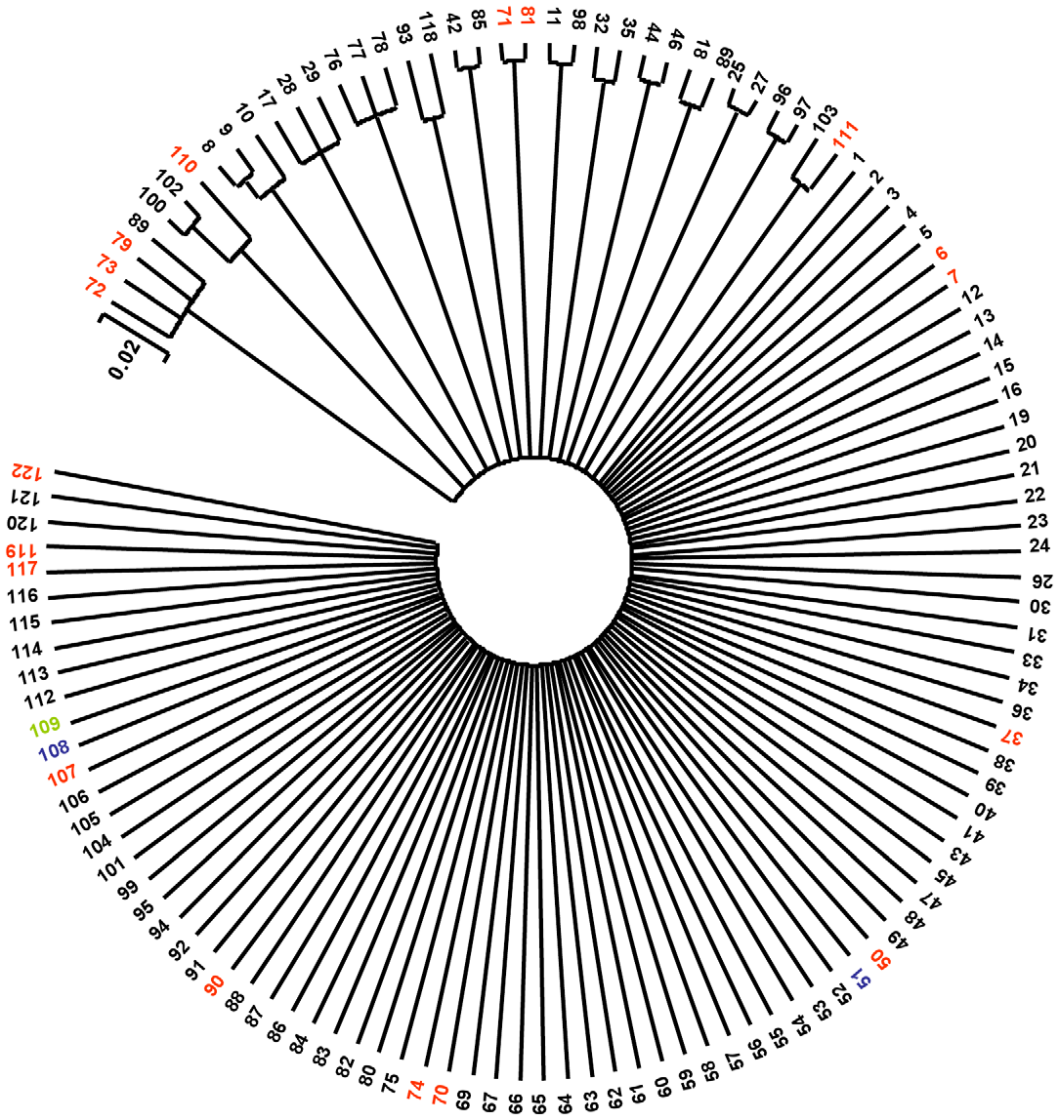
Phylogentic tree of 122 *S. mutans* STs. 50% majority-rule consensus radiant phylogenetic tree derived from 6 trees generated with the *ClonalFrame* program for the 6 housekeeping loci in *S. mutans* and imported into MEGA version 3.1, displaying the clonal relationship between the STs of the *S. mutans* population at 6 loci (*accC, gki, lepA, recP*, *sodA* and *tyrS*).

The ClonalFrame analysis (Figure 2) of the concatenated sequences of the six housekeeping loci confirmed only one of the associations [STs 44 and 46] identified in the eBURST analysis (Figure S3). The majority of STs occurred as singlets with no apparent clonal relationship to each other. The remaining STs formed only 6 doublets and 1 triplet cluster and overall there was no clonal structure. There was no clustering of serotypes within the ClonalFrame consensus tree and there was no evidence of a geographical relationship between STs. When we analysed the distribution of *S. mutans* isolates in the ClonalFrame tree there was no clustering of isolates from different clinical origins, so isolates from active caries lesions were clustered with those from caries-free subjects.

## Discussion

The population of *S. mutans* is characterised by low nucleotide sequence heterogeneity similar to that seen in *Streptococcus agalactiae* [52], but substantially lower than that of other streptococcal populations including *S. salivarius* [29], *S. oralis* [30] and *S. pneumoniae* [24]. The low level of divergence reported here is similar to that reported previously for a different set of gene sequences determined for *S. mutans* isolates primarily recovered from subjects in Japan and Finland [35]. However, despite low divergence a large number of STs was identified. The low level of divergence is unlikely, in the present study, to be a result of biased sampling since we included *S. mutans* isolates recovered from caries-free and caries-active subjects from several geographically distinct communities. The inclusion of putative virulence genes in an MLST scheme is not usual since such schemes are traditionally based on housekeeping genes that are not considered to be under diversifying selective pressure. However, secreted putative virulence genes have been included in MLST schemes in order to maximize the discrimination between strains [34]. Here we found 122 STs in 135 isolates while 92 STs were identified amongst 102 Japanese and Finnish isolates [35] indicating that with different genes a similar rate of ST identification and strain discrimination was observed. The inclusion of virulence genes did not significantly increase strain discrimination.

Of interest is the high level of diversity observed in the sequence of *gtfB* that has previously been discounted for use in MLST schemes [35] since it is believed that the virulence genes of *S. mutans* are not as genetically diverse as those of other organisms. Limited DNA polymorphisms in the 5′ regions of the *gtfB* and *gtfC* genes have been reported and this apparent limited polymorphism was confirmed by analysis of restriction fragment length polymorphisms of *gtfB* [53]. Here however, sequencing around 10% of the *gtfB* we observed 47 polymorphic sites equivalent to 10.32% of all sites in the 455 bp fragment sequenced. This level of polymorphism was greater than that observed in any of the other 7 genes studied here or in any of the 8 genes investigated previously [35]. The other virulence gene we investigated, *spaP* [11% of total length], also exhibited greater polymorphic diversity (7.96%) than 5 of the other 6 genes investigated here and all the loci investigated previously [35]. This observation is in contrast with the earlier reports, based primarily on probe binding and restriction fragment polymorphism analysis, that *spaP* gene in *S. mutans* is highly conserved [54]. Probe binding and RFLP analysis may not be as discriminatory as required and clearly these approaches are not as discriminatory as the method employed here in which the single nucleotide changes were detectable.

The dN/dS values for each locus was <1 which is normally taken to signify that the population was subject to stabilizing selection; nonsynonymous mutations are not selected [55]. However, the ratio dN/dS was originally calculated to compare genetic sequences of independent divergent species and not for the analysis of samples from a single population [56]. Nonetheless, dN/dS has been used widely to analyse MLST data derived from bacterial populations although it has recently been suggested that it may be impossible to infer selective pressure from dN/dS obtained from a single population [57].

The significant negative values calculated for Tajima's D value signifies an excess of low frequency polymorphisms, indicating population size expansion or positive selection. The two virulence factors, *gtfB* and *spaP*, were amongst the most diverse loci investigated but there was no evidence for the positive selection of any amino acid [codon] as determined by the SLAC analysis although for both of these alleles the number of nonsynonymous changes was greater than the number of synonymous changes. However, the r/m ratio was <1 indicating that in these two putative virulence determinants, mutation was the main driver of sequence diversity. Positive selection of amino acids might have been expected in these two secreted proteins as similar surface proteins exhibit high variability and mosaic allele structures with evidence for positive selection mediated by immunogenic pressure [32], [33].

The r/m value for the house-keeping genes, 8.3, was high when compared to the range of such values reported previously but less than the values, 17.2 and 23.1 reported for *S. pyogenes* and *S. pneumoniae*, respectively [51]. It may be that a high level of recombination is characteristic of the housekeeping genes of human-associated streptococci; such recombination will be primarily intra-species with a lower level of inter-species recombination. The majority of STs occurred as singletons so that although there was limited sequence diversity, as evident in very low π and θ values, suggesting that the sequence diversity was magnified by extensive intra-species recombination generating genotypes which can be readily distinguished by sequence analysis [58]. The result appears to be that STs exhibited only a very limited clonal relationship as shown in Fig. 2. The general lack of relationship between STs is in accord with the poorly supported NJ tree meaning the NJ analysis is not necessarily a suitable method for analysing phylogenetic relationships between bacteria using MLST data [50]. In a recent, study clonal complexes were identified in a dendrogram based on concatenated sequences derived from 102 isolates of *S. mutans* with cluster analysis by the unweighted pair group method using arithmetic means [35]. However, the robustness of the tree was not presented as no bootstrapping values were given. Nonetheless it was suggested that serotype *e*, *f*, and *k* strains were present in clonal complexes and as serotype c strains were distributed throughout the tree the ancestral strain of *S. mutans* was serotype *c*.

In this collection of isolates the majority were serotype c with fewer strains identified as serotypes e or f and only 1 isolate, from London UK, was found to be serotype k. All STs with multiple isolates were composed of isolates with the same serotype. In the ClonalFrame analysis five clusters each of 3 STs were identified. Of these 3 were composed of only serotype c isolates while the other 2 clusters were composed of isolates identified as serotype c and e. Of the 9 doublets 7 were composed only of serotype c isolates, one of serotype e isolates and 1 had both serotype e and c isolates. The different distributions of serotypes between STs in the present study and the previous one may be due to the selection of genes used to construct the MLST scheme although in both schemes the overall diversity of allelic sequences was low. The serotype-specific antigens of *S. mutans* are composed of a α1,2- and α1,3-linked rhamnan backbone with glucose α1,2-linked in serotype c isolates, β1,2- and α1,3- in serotypes e and f, respectively. The addition of the glucose units to the rhamnan backbone requires the acquisition of specific transferases and it was not possible to identify any of the three serotypes as the ancestral serotype and none of these serotypes may be the ancestral serotype [12]. For a strain possessing serotype c to be the ancestral *S. mutans* strain the serotype c locus must be lost before either the e or f serotype-specific locus was acquired or strains with two serotype-specific loci must arise prior to the loss of one. Such strains must be rare, if they exist, as none has been previously reported. A study of the genetic relatedness of plasmid strains of *S. mutans* by chromosomal fingerprinting concluded that there was an association between possessing the serotype e locus and plasmid carriage [59]. In the present study we did not examine strains for plasmid carriage as it is found in only 5% of strains but we did find that serotype e isolates were distributed throughout the phylogenetic tree derived using ClonalFrame. These data suggest that strains that possess the serotype c locus are more successful than the other serotypes and that acquisition of a serotype-specific locus has probably occurred numerous times.

The data presented in this study have confirmed that *S. mutans* is a highly diverse species in terms of nucleotide sequence although the overall level of heterogeneity within the sequences of individual loci is low. The driver for heterogeneity in the house-keeping genes was recombination while for the two virulence determinants mutation was the more likely cause of diversity. The MLST approach taken here for the typing of *S. mutans* strains could be employed using the six house keeping loci alone; the inclusion of the virulence determinants had little effect on its ability to discriminate between isolates. There was no convincing evidence of a geographical distribution of individual *S. mutans* clones nor that a *S. mutans* strain possessing the serotype c-specific gene was the ancestral strain of all *S. mutans* though strains bearing serotype c are clearly more abundant and therefore more successful than other *S. mutans* serotypes. The overall low sequence diversity may suggest that *S. mutans* is a newly emerged species which has not existed for sufficiently long to accumulate large numbers of mutations but those that have occurred have been shuffled as a consequence of intra-species recombination generating genotypes which can be readily distinguished by sequence analysis. These results with MLST provide new insights and complement other studies on *S. mutans* diversity using genome sequence comparison [60] or comparative genome hybridization [61], [62].

## Supporting Information

Figure S1 Splitstree analysis of the 8 loci. Only *tyrS* gave a significant PHI value.
(0.11 MB TIF)

Figure S2 Neighbour-joining tree constructed using MEGA v4.0, showing relationships between the concatenated sequences of all *S. mutans* STs (n = 122). Bootstrap values are indicated at corresponding nodes and STs at end of branches. Bar is 0.0005 substitutions per site.
(0.06 MB TIF)

Figure S3 Radial diagram of the *eBURST* analysis of *S. mutans* STs.
(0.07 MB TIF)

Table S1 Sequence types (ST) and allelic profiles; with N, number of ST occurrences.
(0.36 MB DOC)
