# Supplementary figures and images for: Generation of Diversity in Streptococcus mutans Genes Demonstrated by MLST
Source: PLoS One. 2010 Feb 5;5(2):e9073. doi: 10.1371/journal.pone.0009073 (PMC2816709; doi:10.1371/journal.pone.0009073)

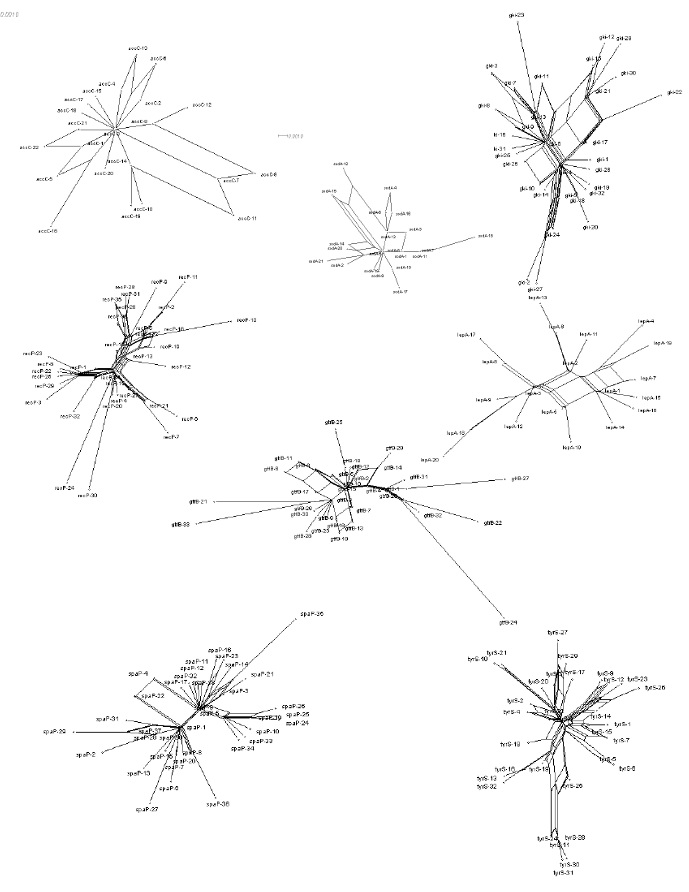

Supplement: Figure S1 — Splitstree analysis of the 8 loci. Only tyrS gave a significant PHI value. (0.11 MB TIF) [file pone.0009073.s001.tif]

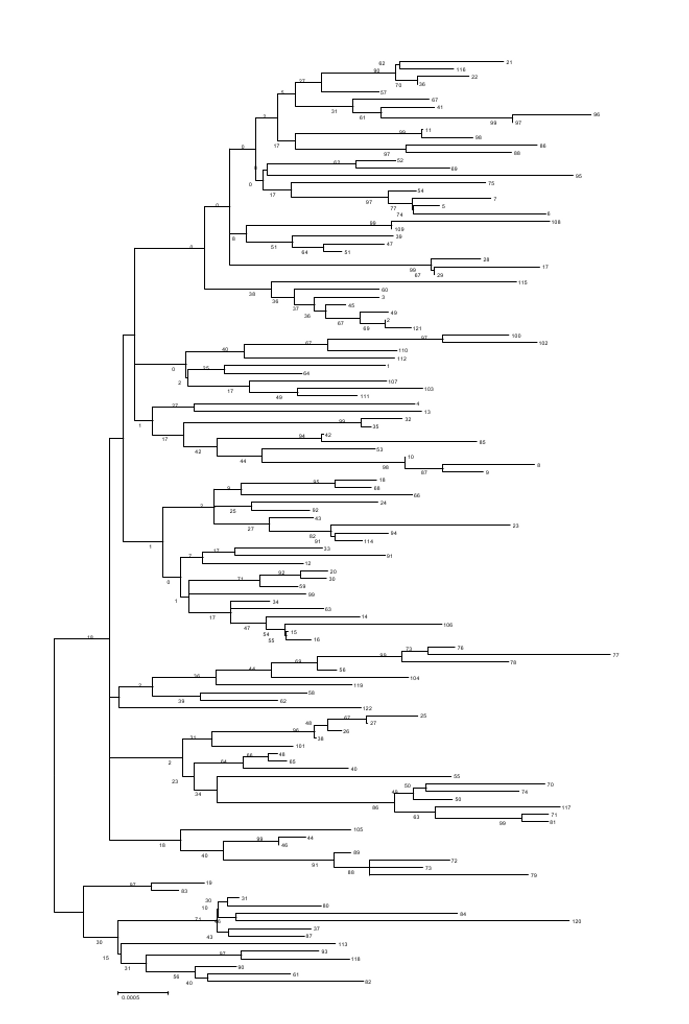

Supplement: Figure S2 — Neighbour-joining tree constructed using MEGA v4.0, showing relationships between the concatenated sequences of all S. mutans STs (n = 122). Bootstrap values are indicated at corresponding nodes and STs at end of branches. Bar is 0.0005 substitutions per site. (0.06 MB TIF) [file pone.0009073.s002.tif]

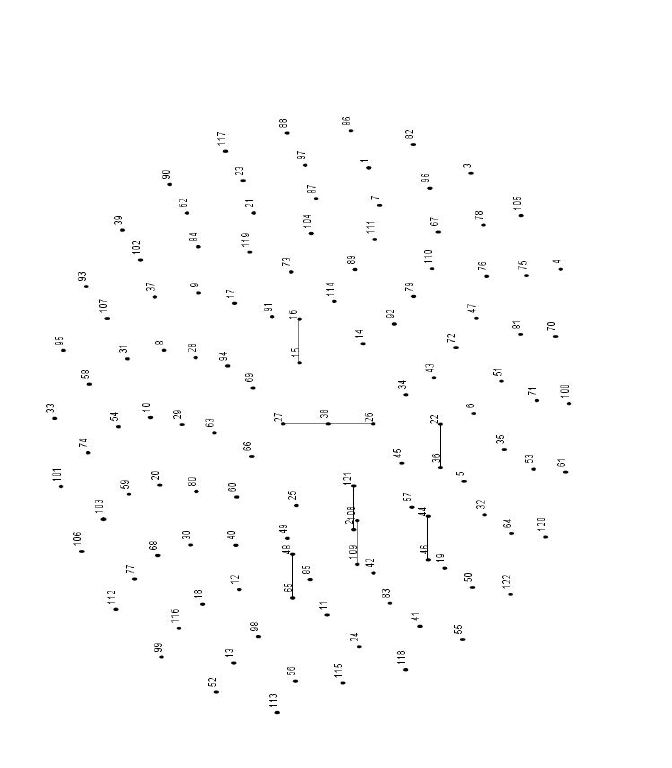

Supplement: Figure S3 — Radial diagram of the eBURST analysis of S. mutans STs. (0.07 MB TIF) [file pone.0009073.s003.tif]
