# Supplementary material for: Generation of Diversity in Streptococcus mutans Genes Demonstrated by MLST
Source: PLoS One. 2010 Feb 5;5(2):e9073. doi: 10.1371/journal.pone.0009073 (PMC2816709; doi:10.1371/journal.pone.0009073)
Supplement: Table S1 — Sequence types (ST) and allelic profiles; with N, number of ST occurrences. (0.36 MB DOC) [file pone.0009073.s004.doc]

**Supplementary Table 1.** Sequence types (ST) and allelic profiles; with N, number of ST occurrences

| **Strain** | **ST** | ***accC*** | ***gki*** | ***lepA*** | ***recP*** | ***sodA*** | ***tyrS*** | ***gtfB*** | ***spaP*** | **Clinical and geographical source** | **Year** | **Serotype** |
| --- | --- | --- | --- | --- | --- | --- | --- | --- | --- | --- | --- | --- |
| F5M4 | 1 | 1 | 4 | 13 | 1 | 17 | 3 | 9 | 13 | Dental plaque Newcastle, UK | 1994 | c |
| 60 | 2 | 1 | 1 | 1 | 1 | 1 | 1 | 1 | 1 | Dental plaque, Newcastle UK | 1997 | c |
| FSM2 | 2 | 1 | 1 | 1 | 1 | 1 | 1 | 1 | 1 | Dental plaque Newcastle, UK | 1994 | c |
| LT11 | 2 | 1 | 1 | 1 | 1 | 1 | 1 | 1 | 1 | Dental plaque, Farmington, USA | 1993 | c |
| VAB | 3 | 1 | 4 | 1 | 11 | 14 | 1 | 1 | 1 | Dental plaque, Virginia, USA | 1986 | c |
| U138 | 4 | 1 | 20 | 2 | 1 | 18 | 6 | 18 | 20 | Caries free supra-gingival plaque, UK | 2006 | c |
| 1738 | 5 | 1 | 6 | 1 | 15 | 27 | 5 | 1 | 3 | Caries free subgingival plaque, UK | 2006 | c |
| 24 | 6 | 1 | 6 | 27 | 1 | 27 | 5 | 1 | 3 | Caries active, Iceland | 1990 | c |
| Y | 7 | 1 | 6 | 1 | 1 | 27 | 21 | 37 | 3 | Caries active, Iceland | 1990 | c |
| G128 | 8 | 2 | 24 | 1 | 19 | 5 | 3 | 3 | 3 | Caries free supra-gingival plaque, UK | 2006 | c |
| G132 | 9 | 2 | 2 | 1 | 2 | 2 | 3 | 3 | 3 | Caries free supra-gingival plaque UK | 2006 | c |
| G140 | 10 | 2 | 5 | 1 | 2 | 5 | 3 | 3 | 3 | Caries free supra-gingival plaque UK | 2006 | c |
| N29 | 11 | 2 | 6 | 2 | 6 | 1 | 5 | 8 | 5 | Dental plaque, London UK | 1991 | c |
| MT4863 | 12 | 2 | 6 | 3 | 1 | 22 | 6 | 9 | 14 | Dental plaque Osaka, Japan | 1980 | c |
| A13 | 13 | 3 | 1 | 2 | 7 | 10 | 6 | 1 | 7 | Dentine 'leathery' root caries lesion, UK | 2006 | c |
| A19 | 14 | 3 | 13 | 6 | 1 | 1 | 6 | 1 | 1 | Dentine 'leathery' root caries lesion, UK | 2006 | c |
| A17 | 15 | 3 | 16 | 3 | 1 | 1 | 6 | 1 | 1 | Dentine 'leathery' root caries lesion, UK | 2006 | c |
| A24 | 15 | 3 | 16 | 3 | 1 | 1 | 6 | 1 | 1 | Dentine 'leathery' root caries lesion, UK | 2006 | c |
| A25 | 15 | 3 | 16 | 3 | 1 | 1 | 6 | 1 | 1 | Dentine 'leathery' root caries lesion,UK | 2006 | c |
| A30 | 16 | 3 | 16 | 17 | 1 | 1 | 6 | 1 | 1 | Dentine 'leathery' root caries lesion, UK | 2006 | c |
| A7 | 17 | 3 | 21 | 1 | 13 | 1 | 5 | 9 | 9 | Dentine 'leathery' root caries lesion, UK | 2006 | c |
| A9 | 18 | 3 | 23 | 3 | 3 | 5 | 16 | 1 | 1 | Dentine 'leathery' root caries lesion, UK | 2006 | c |
| G123 | 19 | 3 | 4 | 3 | 17 | 13 | 5 | 9 | 25 | Caries free supra-gingival plaque, UK | 2006 | c |
| G138 | 20 | 3 | 4 | 3 | 3 | 4 | 5 | 4 | 1 | Caries free supra-gingival plaque, UK | 2006 | c |
| M19 | 21 | 3 | 6 | 4 | 4 | 6 | 6 | 5 | 5 | Plaque active occlusal caries, UK | 2006 | c |
| M21 | 22 | 3 | 6 | 1 | 1 | 7 | 6 | 5 | 5 | Plaque active occlusal caries UK | 2006 | c |
| T4 | 23 | 3 | 8 | 6 | 1 | 9 | 7 | 9 | 1 | Caries free supra-gingival plaque, UK | 2006 | c |
| T5 | 24 | 3 | 9 | 3 | 1 | 11 | 6 | 9 | 8 | Caries free supra-gingival plaque, UK | 2006 | c |
| U134 | 25 | 3 | 4 | 7 | 8 | 12 | 8 | 10 | 5 | Dentine 'leathery' root caries lesion, UK | 2006 | c |
| LML6 | 26 | 3 | 4 | 2 | 8 | 13 | 8 | 10 | 5 | Dental plaque, Newcastle, UK | 2001 | c |
| U135 | 26 | 3 | 4 | 2 | 8 | 13 | 8 | 10 | 5 | Dentine 'leathery' root caries lesion, UK | 2006 | c |
| U136 | 27 | 3 | 4 | 2 | 3 | 12 | 8 | 10 | 5 | Dentine 'leathery' root caries lesion, UK | 2006 | c |
| W7 | 28 | 3 | 10 | 1 | 6 | 1 | 5 | 11 | 9 | Supra-gingival plaque caries lesion, UK | 2006 | c |
| N28 | 29 | 3 | 12 | 1 | 6 | 1 | 5 | 9 | 9 | Dental plaque, Newcastle, UK | 1997 | c |
| SM5 | 29 | 3 | 12 | 1 | 6 | 1 | 5 | 9 | 9 | Supra-gingival plaque, Hong Kong | 2001 | c |
| 34 | 30 | 3 | 4 | 9 | 3 | 15 | 5 | 4 | 1 | Dental plaque, Newcastle, UK | 1997 | c |
| 35 | 31 | 3 | 4 | 10 | 3 | 16 | 5 | 5 | 11 | Dental plaque, Newcastle, UK | 1997 | c |
| FSM1 | 32 | 3 | 14 | 4 | 3 | 18 | 9 | 13 | 14 | Dental plaque, Newcastle, UK | 1994 | c |
| FSM5 | 33 | 3 | 12 | 3 | 1 | 1 | 1 | 14 | 15 | Dental plaque, Newcastle, UK | 1994 | c |
| GS5 | 34 | 3 | 6 | 3 | 1 | 1 | 5 | 1 | 1 | Dental plaque, Boston, USA | 1966 | c |
| ST8 | 34 | 3 | 6 | 3 | 1 | 1 | 5 | 1 | 1 | Caries free saliva, UK | 2006 | c |
| Ingbritt | 35 | 3 | 6 | 4 | 3 | 19 | 9 | 13 | 14 | Dental plaque, Malmo, Sweden | 1966 | c |
| LML2 | 36 | 3 | 6 | 1 | 1 | 21 | 6 | 5 | 5 | Dental plaque, Newcastle, UK | 2001 | c |
| LML4 | 37 | 3 | 4 | 8 | 9 | 17 | 5 | 5 | 11 | Dental plaque, Newcastle, UK | 2001 | e |
| LML5 | 38 | 3 | 4 | 2 | 3 | 13 | 8 | 10 | 5 | Dental plaque, Newcastle, UK | 2001 | c |
| LML7 | 39 | 3 | 17 | 14 | 3 | 16 | 5 | 9 | 18 | Dental plaque, Newcastle, UK | 2001 | c |
| LML9 | 40 | 3 | 4 | 15 | 10 | 17 | 5 | 16 | 14 | Dental plaque, Newcastle, UK | 2001 | c |
| M24 | 41 | 3 | 12 | 1 | 3 | 1 | 5 | 17 | 5 | Plaque active occlusal caries, UK | 2006 | c |
| M2A | 42 | 3 | 6 | 4 | 3 | 5 | 10 | 9 | 17 | Plaque active occlusal caries, UK | 2006 | c |
| M40 | 43 | 3 | 4 | 3 | 3 | 17 | 7 | 9 | 1 | Plaque active occlusal caries, UK | 2006 | c |
| 3209 | 44 | 3 | 1 | 3 | 1 | 25 | 5 | 9 | 17 | Dental plaque, Birmingham, UK | 1979 | c |
| 66 | 45 | 3 | 6 | 1 | 1 | 1 | 1 | 1 | 1 | Dental plaque, Newcastle, UK | 1997 | c |
| LML3 | 45 | 3 | 6 | 1 | 1 | 1 | 1 | 1 | 1 | Dental plaque, Newcastle, UK | 2001 | c |
| T2A | 45 | 3 | 6 | 1 | 1 | 1 | 1 | 1 | 1 | Caries free supra-gingival plaque,UK | 2006 | c |
| NG8 | 46 | 3 | 1 | 3 | 1 | 1 | 5 | 9 | 17 | Dental plaque, New Guinea | 1970 | c |
| V1996 | 47 | 3 | 4 | 14 | 12 | 15 | 5 | 1 | 19 | Dental plaque, Virginia, USA | 1991 | c |
| V403 | 47 | 3 | 4 | 14 | 12 | 15 | 5 | 1 | 19 | Dental plaque, Virginia, USA | 1986 | c |
| U139 | 48 | 3 | 4 | 15 | 3 | 13 | 5 | 5 | 14 | Caries free supra-gingival plaque, UK | 2006 | c |
| U2B | 49 | 3 | 1 | 1 | 14 | 1 | 1 | 1 | 1 | Dental plaque, Turkey | 2005 | c |
| W6 | 50 | 3 | 1 | 15 | 3 | 26 | 12 | 19 | 8 | Supra-gingival plaque caries lesion, UK | 2006 | e |
| 1442 | 51 | 3 | 4 | 14 | 2 | 15 | 6 | 1 | 19 | Caries free subgingival plaque, UK | 2006 | c |
| F1B | 52 | 3 | 4 | 4 | 3 | 1 | 2 | 2 | 22 | Supra-gingival plaque caries lesion, UK | 2005 | f |
| H1B | 53 | 3 | 22 | 14 | 3 | 5 | 13 | 9 | 17 | Dental plaque, Turkey | 2006 | c |
| O2A | 54 | 3 | 6 | 1 | 1 | 27 | 14 | 1 | 3 | Dental plaque, Turkey | 2005 | c |
| S1B | 55 | 3 | 1 | 15 | 1 | 15 | 15 | 21 | 23 | Supra-gingival plaque, San Francisco | 2001 | c |
| SF1 | 56 | 3 | 6 | 3 | 1 | 14 | 17 | 2 | 16 | Supra-gingival plaque, San Francisco | 2001 | c |
| SF12 | 57 | 3 | 4 | 1 | 1 | 1 | 6 | 5 | 5 | Supra-gingival plaque, San Francisco | 2001 | c |
| SF14 | 58 | 3 | 4 | 18 | 16 | 15 | 10 | 2 | 5 | Supra-gingival plaque, San Francisco | 2001 | c |
| SF8 | 59 | 3 | 4 | 3 | 1 | 15 | 5 | 22 | 1 | Supra-gingival plaque, San Francisco | 2001 | c |
| SM2 | 60 | 3 | 6 | 1 | 1 | 14 | 1 | 23 | 1 | Supra-gingival plaque, Hong Kong | 2001 | c |
| SM3 | 60 | 3 | 6 | 1 | 1 | 14 | 1 | 23 | 1 | Supra-gingival plaque, Hong Kong | 2001 | c |
| SM4 | 61 | 3 | 4 | 15 | 6 | 18 | 1 | 1 | 11 | Supra-gingival plaque, Hong Kong | 2001 | c |
| SM6 | 62 | 3 | 4 | 18 | 1 | 15 | 11 | 5 | 5 | Supra-gingival plaque, Hong Kong | 2001 | c |
| ST1 | 63 | 3 | 6 | 19 | 6 | 1 | 6 | 1 | 15 | Caries free saliva, UK | 2006 | c |
| ST3 | 64 | 3 | 4 | 7 | 1 | 17 | 11 | 9 | 1 | Caries free saliva, UK | 2006 | c |
| ST4 | 65 | 3 | 4 | 15 | 3 | 17 | 5 | 5 | 14 | Caries free saliva, UK | 2006 | c |
| ST5 | 66 | 3 | 12 | 3 | 18 | 17 | 1 | 9 | 26 | Caries free saliva, UK | 2006 | c |
| ST6 | 67 | 3 | 12 | 1 | 1 | 1 | 16 | 9 | 5 | Caries free saliva, UK | 2006 | c |
| ST7 | 68 | 3 | 4 | 3 | 8 | 5 | 16 | 1 | 1 | Caries free saliva, UK | 2006 | c |
| U2A | 69 | 3 | 1 | 1 | 1 | 1 | 2 | 2 | 2 | Dental plaque, Turkey | 2005 | c |
| 14A | 70 | 3 | 4 | 25 | 3 | 26 | 12 | 24 | 17 | Caries free, Iceland | 1990 | e |
| 14D | 71 | 3 | 4 | 15 | 3 | 26 | 12 | 25 | 11 | Caries free, Iceland | 1990 | e |
| 20 | 72 | 3 | 1 | 23 | 1 | 1 | 18 | 9 | 27 | Caries active, Iceland | 1990 | e |
| 21 | 73 | 3 | 1 | 26 | 1 | 29 | 7 | 9 | 27 | Caries active, Iceland | 1990 | e |
| 28 | 74 | 3 | 4 | 15 | 3 | 35 | 12 | 26 | 28 | Caries active, Iceland | 1990 | e |
| 43 | 75 | 3 | 25 | 1 | 1 | 1 | 6 | 27 | 28 | Caries active, Iceland | 1990 | c |
| 66_2 | 76 | 3 | 26 | 3 | 1 | 20 | 17 | 28 | 16 | Caries free, Iceland | 1990 | c |
| 66_2A | 77 | 3 | 19 | 3 | 1 | 30 | 17 | 29 | 16 | Caries free, Iceland | 1990 | c |
| 66_2B | 78 | 3 | 27 | 28 | 1 | 20 | 17 | 30 | 16 | Caries free, Iceland | 1990 | c |
| 66’ | 79 | 3 | 1 | 21 | 1 | 1 | 7 | 9 | 27 | Caries free, Iceland | 1990 | e |
| 77 | 80 | 3 | 4 | 29 | 3 | 17 | 5 | 5 | 29 | Caries free, Iceland | 1990 | c |
| 8E | 81 | 3 | 4 | 15 | 3 | 31 | 12 | 19 | 11 | Caries free, Iceland | 1990 | e |
| 98 | 82 | 3 | 12 | 3 | 20 | 18 | 1 | 1 | 11 | Caries active, Iceland | 1990 | c |
| A | 83 | 3 | 4 | 3 | 3 | 13 | 5 | 32 | 25 | Caries active, Iceland | 1990 | c |
| B | 84 | 3 | 4 | 33 | 3 | 17 | 5 | 33 | 11 | Caries active, Iceland | 1990 | c |
| BP | 85 | 3 | 9 | 22 | 3 | 5 | 10 | 9 | 17 | Caries free, Iceland | 1990 | c |
| C | 86 | 3 | 28 | 20 | 3 | 32 | 19 | 31 | 5 | Caries active, Iceland | 1990 | c |
| F | 87 | 3 | 29 | 15 | 3 | 33 | 5 | 5 | 11 | Caries active, Iceland | 1990 | c |
| SVH | 88 | 3 | 6 | 32 | 3 | 34 | 19 | 36 | 5 | Caries free, Iceland | 1990 | c |
| 04_27 | 89 | 3 | 1 | 3 | 1 | 15 | 7 | 9 | 27 | Brazil, caries-free saliva | 2007 | c |
| 04_13 | 90 | 3 | 6 | 2 | 1 | 18 | 6 | 1 | 11 | Brazil, caries-free saliva | 2007 | e |
| 04_08 | 91 | 3 | 9 | 3 | 1 | 37 | 7 | 9 | 1 | Brazil, caries-free saliva | 2007 | c |
| 04_37 | 92 | 3 | 9 | 3 | 1 | 17 | 5 | 9 | 1 | Brazil, caries-free saliva | 2007 | c |
| 04_01 | 93 | 3 | 12 | 30 | 1 | 36 | 5 | 1 | 31 | Brazil, caries-free saliva | 2007 | c |
| 04_44 | 94 | 3 | 31 | 3 | 1 | 17 | 7 | 9 | 1 | Brazil, caries-free saliva | 2007 | c |
| G133 | 95 | 4 | 3 | 2 | 1 | 3 | 4 | 2 | 4 | Caries free supra-gingival plaque, UK | 2006 | c |
| M38 | 96 | 5 | 7 | 1 | 5 | 8 | 5 | 6 | 6 | Plaque active occlusal caries, UK | 2006 | c |
| M39 | 97 | 5 | 7 | 1 | 5 | 1 | 5 | 7 | 5 | Plaque active occlusal caries, UK | 2006 | c |
| T11 | 98 | 6 | 6 | 5 | 6 | 1 | 5 | 8 | 5 | Caries free supra-gingival plaque, UK | 2006 | c |
| NCTC10449 | 99 | 7 | 4 | 3 | 2 | 14 | 1 | 1 | 1 | Dental plaque, London, UK | 1972 | c |
| 12 | 100 | 8 | 11 | 8 | 3 | 13 | 3 | 12 | 10 | Dental plaque, London, UK | 1992 | c |
| LML8 | 101 | 8 | 4 | 2 | 3 | 15 | 4 | 9 | 8 | Dental plaque, Newcastle, UK | 2002 | c |
| AT1O | 102 | 9 | 15 | 11 | 3 | 13 | 3 | 12 | 10 | Dental plaque, Malmo, Sweden | 1968 | c |
| B2 | 103 | 10 | 15 | 12 | 1 | 13 | 3 | 12 | 12 | Dental plaque, Malmo, Sweden | 1968 | c |
| ML1 | 103 | 10 | 15 | 12 | 1 | 13 | 3 | 12 | 12 | Dental plaque, Newcastle, UK | 2001 | c |
| L13 | 104 | 11 | 15 | 3 | 1 | 20 | 10 | 5 | 16 | Dental plaque, Heksinki, Finland | 2000 | c |
| L18 | 105 | 12 | 4 | 3 | 3 | 15 | 11 | 15 | 17 | Dental plaque, Heksinki, Finland | 2000 | c |
| A27 | 106 | 13 | 18 | 16 | 1 | 23 | 6 | 1 | 1 | Dentine 'leathery' root caries lesion, UK | 2006 | c |
| 13 | 107 | 14 | 19 | 12 | 1 | 13 | 3 | 12 | 1 | Dental plaque, Newcastle, UK | 1995 | e |
| 19 | 108 | 15 | 12 | 14 | 1 | 24 | 5 | 1 | 5 | Dental plaque, London, UK | 1991 | f |
| 21 | 109 | 15 | 12 | 14 | 1 | 15 | 5 | 1 | 5 | Dental plaque, London, UK | 1991 | k |
| E | 110 | 15 | 15 | 2 | 1 | 15 | 3 | 12 | 10 | Caries active, Iceland | 1990 | e |
| A51 | 110 | 15 | 15 | 2 | 1 | 15 | 3 | 12 | 10 | Dental plaque, Newcastle, UK | 1995 | c |
| MT4653 | 111 | 15 | 15 | 2 | 1 | 15 | 3 | 5 | 12 | Dental plaque, Osaka, Japan | 1980 | e |
| 1151 | 112 | 15 | 4 | 18 | 3 | 13 | 5 | 20 | 21 | Caries free subgingival plaque, UK | 2006 | c |
| SA38 | 113 | 16 | 5 | 3 | 3 | 15 | 11 | 1 | 24 | Supra-gingival plaque, South Africa | 2001 | c |
| SA41 | 114 | 17 | 9 | 3 | 1 | 17 | 7 | 9 | 1 | Supra-gingival plaque, South Africa | 2001 | c |
| P | 115 | 17 | 1 | 31 | 1 | 14 | 10 | 35 | 1 | Caries active, Iceland | 1990 | c |
| SM1 | 116 | 18 | 6 | 4 | 16 | 21 | 6 | 5 | 5 | Dental plaque, London, UK | 2001 | c |
| 14B | 117 | 19 | 4 | 15 | 3 | 28 | 12 | 19 | 16 | Caries free, Iceland | 1990 | e |
| 99 | 118 | 20 | 6 | 30 | 1 | 14 | 5 | 31 | 30 | Caries active, Iceland | 1990 | c |
| AE | 119 | 21 | 15 | 3 | 1 | 20 | 10 | 5 | 32 | Caries active, Iceland | 1990 | e |
| D | 120 | 21 | 4 | 24 | 3 | 17 | 20 | 34 | 11 | Caries active, Iceland | 1990 | c |
| Z | 121 | 22 | 1 | 1 | 1 | 1 | 1 | 1 | 1 | Caries active, Iceland | 1990 | c |
| 04_09 | 122 | 23 | 30 | 34 | 1 | 13 | 6 | 1 | 32 | Brazil, caries-free saliva | 2007 | e |
